# Identification and a culture method for a *Helicotylenchus microlobus* from tomato in China

**DOI:** 10.1186/s40850-022-00144-7

**Published:** 2022-07-25

**Authors:** Yan-Hui Xia, Jin Li, Fei-Fei Xu, Bin Lei, Hong-Lian Li, Ke Wang, Yu Li

**Affiliations:** 1 College of Plant Protection, Henan Agricultural University, Zhengzhou, China; 2 National Key Laboratory of Wheat and Maize Crop Science, Henan Agricultural University, Zhengzhou, China; 3 Institute of Nuclear Technology and Biotechnology, Key Laboratory of Crop Ecophysiology and Farming System in Desert Oasis Region, Ministry of Agriculture and Rural Affairs, Xinjiang Academy of Agricultural Sciences, Urumqi, China; 4 Institute of Nuclear Technology and Biotechnology, Xinjiang Academy of Agricultural Sciences/Xinjiang Crop Chemical Control Engineering Technology Research Center, Urumqi, China

**Keywords:** In vitro culture, *Helicotylenchus microlobus*, Identification, Reproduction, Tomato

## Abstract

**Background:**

The nematodes of the genus *Helicotylenchus* are root parasites of a wide variety of plants, and certain species can cause serious damage to their hosts. During a survey of the plant-parasitic nematode associated with tomato, a population of *Helicotylenchus* was collected from tomato roots and soil samples. Thus, one of the objectives of the study was to confirm the specie of *Helicotylenchus* obtained from the tomato samples based on morphological and molecular characteristics. In addition, a mass pure culture of plant-parasitic nematodes is key to pathogenicity studies and many other biological studies. However, a successful mass rearing method for *Helicotylenchus* has not been reported. Thus, the other objective of the study was to establish a method of culturing *Helicotylenchus*.

**Results:**

Based on both the morphological characteristics and molecular analysis of the internal transcribed spacer (ITS) and D2-D3 expansion region of 28S ribosomal RNA (rRNA) sequences the specimens were identified as *Helicotylenchus microlobus*. Phylogenetic analysis with the rRNA sequences of the ITS and 28S D2-D3 regions was consistent with molecular identification, suggesting this population formed a highly supported clade with other *H. microlobus* populations. Additionally, a method for culture of *H. microlobus* on carrot disks was established, and the effect of temperature on the reproduction rate (Rr) of *H. microlobus* was investigated. The optimum temperature for culturing *H. microlobus* on carrot disks was 27.5 °C and, after inoculation with 30 females of *H. microlobus* at 27.5 °C for 90 days, Rr reached 406.

**Conclusions:**

To our knowledge, this is the first detailed description of *H. microlobus* from tomato in China. This study also demonstrated that the carrot disk method is suitable for the culture of *H. microlobus*. This study lays a foundation for other related research on *H. microlobus*, and has significance for the study of *Helicotylenchus.*

## Background

Tomato (*Solanum lycopersicum* L.) is one of the most economically important members of the family Solanaceae and is cultivated worldwide [1]. It is one of the most important vegetable crops in China and is rich in minerals, vitamins, antioxidants and other micronutrients [2]. China has the largest area of tomato cultivation in the world, and Henan Province is the dominant production area for tomato cultivation [3]. Many plant pathogens can infect tomato, and nematodes perhaps are one of the important pathogens limiting tomato production worldwide and can cause severe economic losses [4].

The genus *Helicotylenchus* Steiner 1945 is considered one of the ten most important plant parasitic nematodes in the world [5]. Within the genus *Helicotylenchus*, more than 200 nominal species have been described worldwide [6]. The genus *Helicotylenchus* is classified as semi-endoparasitic that may occur in large numbers, causing plant growth reduction [7, 8]. After infection by *Helicotylenchus*, plants of outer layer of cortical tissue and the root system were damaged, further resulting in reduced ability of plants to absorb water and nutrients. For example, *Helicotylenchus* species associated with Musaceae damaged the cortical tissue of roots, and reduced the ability of roots for the uptake of water and nutrients [9]. *H. microlobus* infected *Paspalum vaginatum* causing brown lesions on their roots [10]; *H. multicinctus*, *H. dihystera* and *H. erythrinae* have been found to be harmful to banana and plantain crops around the world [9]. Inoculating *H. dihystera* on Olive seedlings caused 78% reduction in weight, and the development of the lateral roots occurred retardation [11]. Some studies suggests that *H. pseudorobustus* feeding in the cortical parenchyma of corn and soybean roots and causing serious damage [8, 12]. At present, 50 *Helicotylenchus* species are reported from China, and these species are reported from a variety of plants including ornamental plants, fruit trees, cucumber, rice, grape and pomegranate [13–15]. However, only *H. dihystera* is currently reported associated with tomatoes in China [16, 17]. At present, *H. microlobus* are not reported from tomato in China. Subbotin et al. recorded that *H. microlobus* was collected in California, Illinois, and Iowa, in the United States of America and in several European countries, including Spain, Italy and Russia [18]; Yan et al. reported *H. microlobus* infecting soybean in North Dakota [19]. Abraham and Dong reported *H. microlobus* in turfgrass in Korea [20].

In addition, a mass pure culture of plant-parasitic nematodes is key to pathogenicity studies and many other biological studies [21]. For most species of plant- parasitic nematodes, establishing a rapid and efficient culture method is a challenge. At present, only a small percentage of plant-parasitic nematodes can be successful cultured. The successfully establishment of an efficient culture method for *Helicotylenchus* has not been reported, and this increased the difficulty to study the pathogenicity and some biology of *Helicotylenchus*.

In 2019, during a survey of the plant-parasitic nematode associated with tomato, a population of *Helicotylenchus* was collected from tomato roots and soil samples in Zhuma village, Tongxu County of Kaifeng city, Henan Province, China. The objectives of this study were to confirm the specie of *Helicotylenchus* obtained from the tomato samples based on morphological and molecular characteristics and establish a method of culturing *Helicotylenchus* on carrot disks.

## Results

### *Helicotylenchus microlobus* Perry in Perry, Darling and Thorne, 1959

**Fig. 1:**
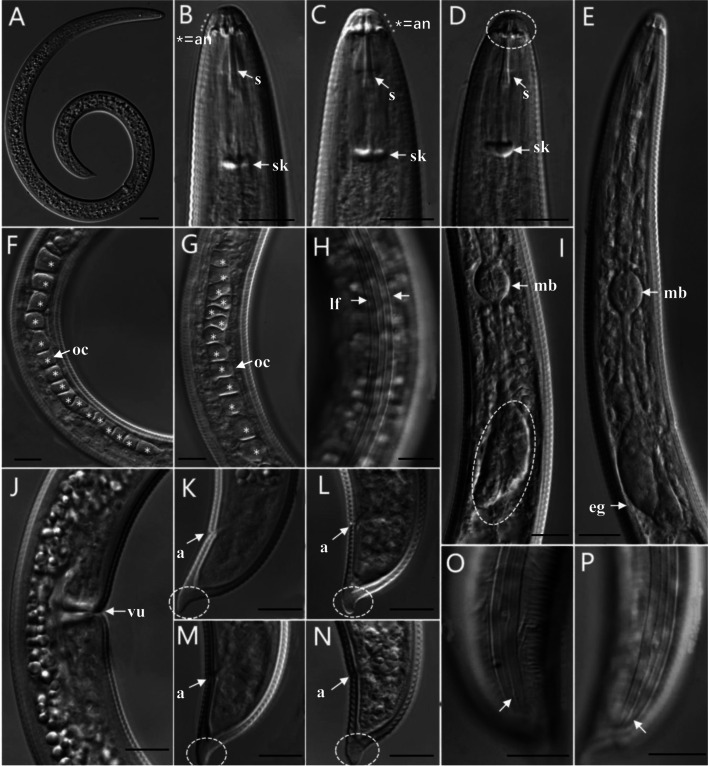
Light micrographs of *Helicotylenchus microlobus* female. **A** Entire body, **B-D** Lip region, e Anterior region, **F-G** Two genital branches, **H** Lateral lines, **I** The junction of genital gland and intestine, **J** Vulval region, **K-N** Tail region, **O-P** Lateral field at tail region. Scale bars: 50 µm (**A**) and 10 µm (**B-P**); an = annuli; s = stylet; sk = stylet knob; oc = ovary cells; lf = lateral field; mb = median bulb; eg = esophageal glands; vu = vulval; a = anal

The *Helicotylenchus* individual population collected from tomato was photographed (Fig. 1). The morphometric data from the population closely resembled *H. microlobus* as described previously [18, 19, 22] (Table 1). Distance between dorsal esophageal gland opening and stylet knobs.

**Table 1:** Morphometrics of females of *Helicotylenchus microlobus*

Character	Populations analyzed in this study	*H. microlobus*				*H. pseudorobustus* Subbotin et al
		Mwamula, et al	Subbotin et al. China	Subbotin et al. USA	Yan et al	
n	18	12	6	12	15	7
L	679.7 ± 36.2(623.1–741.7)	732.5 ± 31.1(680.0–792.9)	622 ± 55.5(555–687)	739 ± 44.5(657–795)	708.5(600–812)	734 ± 53.2(675–817)
a	26.8 ± 0.7(25.6–28.1)	25.6 ± 0.9(24.4–26.9)	25.9 ± 1.2 (24.7–27.5)	26.6 ± 1.1(24.8–28.5)	25.0(21.4–27.1)	27.2 ± 1.6(25.2929.7)
b	5.3 ± 0.6(4.6–6.3)	5.9 ± 0.3(5.4–6.4)	4.7 ± 0.5 (4.4–5.3)	5.2 ± 0.2(4.7–5.5)	5.0(4.4–5.7)	6.0 ± 0.6(5.4–6.9)
b’	4.7 ± 0.4(4.2–5.5)	5.0 ± 0.2(4.6–5.3)	__	__	__	__
c	33.1 ± 2.8(28.2–38.3)	37.7 ± 3.1(31.9–42.9)	37.9 ± 4.8 (32.9–47)	34.8 ± 2.0(32.1–38.9)	35.4(30.2–41.7)	41.4 ± 4.6(35.1–46.2)
c’	1.5 ± 0.2(1.3–1.8)	1.2 ± 0.1(1.1–1.3)	1.2 ± 0.2 (0.9–1.4)	1.3 ± 0.1(1.2–1.5)	1.3(1.0–1.6)	1.1 ± 0.1(1.0–1.3)
V	62.3 ± 2.3(58.3–68.7)	60.5 ± 1.1(58.6–62)	63.2 ± 1.7 (61.8–66.2)	62.6 ± 1.4(60.4–64.8)	61.8(60.0–63.7)	62.6 ± 1.5(60.3–65.3)
O	41.3 ± 2.4(34.5–47.8)	43.2 ± 3.2(38.5–48.2)	__	__	__	__
m	48.8 ± 3.6(44.8–53.4)	43.7 ± 1.6(41.9–47.3)	__	__	__	__
Stylet length	25.6 ± 1.0(25.3–28.8)	25.2 ± 0.8(24.1–26.6)	25.3 ± 0.4 (25.0–25.5)	28.9 ± 0.7 (27.5–30.0)	27.6(26.0–29.0)	27.3 ± 0.8(26.5–29.0)
Conus length	13.0 ± 0.8(11.8–14.6)	11.0 ± 0.6(10.1–11.9)	__	13.2 ± 0.4 (12.5–14.0)	__	13 ± 0.4(12.5–14.0)
DGO	10.9 ± 0.8(9.3–12.5)	10.9 ± 0.8(9.7–12.0)	__	__	__	__
Anterior to median bulb length	78.0 ± 2.3(74.4–88.2)	81.5 ± 3.2(9.7–12.0)	58.0 ± 1.2 (56.9–59	67.3 ± 2.1(64.7–71.8)	__	87.5 ± 5.5(68.8–93.8)
Anterior end to excretory pore	110.0 ± 5.0(103.6–126.7)	80.9 ± 3.1(76.6–86.6)	100 ± 6.3 (94–109)	128 ± 6.3(114–138)	__	111 ± 3.9(105–115)
Pharynx length	144.3 ± 6.0(134.5–155.6)	123.6 ± 3.9(117.6–128.3)	128 ± 3.1 (125–131)	140 ± 6.0(125–145)	142.5(130.0–152.0)	144 ± 8.7(136–156)
Max body diam	25.4 ± 1.5 (23.7–28.7)	28.6 ± 0.8(27.3–29.6)	23.9 ± 1.7 (22.5–26.5)	27.8 ± 1.2(26.0–30.0)	27.3(25.0–33.0)	26.9 ± 0.9(25.0–27.5)
Vulval body diam	23.5 ± 1.9(20.2–27.5)	__	__	__	__	__
Anal body diam	14.3 ± 1.2(11.5–16.4)	16.0 ± 1.0(14.6–17.9)	14.2 ± 0.6 (14.0–15.0	16.0 ± 1.0(15.0–17.5)	15.8(14.0–17.5)	15.7 ± 0.7(14.0–16.0)
Tail length	20.7 ± 1.5(18.2–23.3)	19.5 ± 1.8(16.9–23.2)	17.0 ± 2.2 (12.5–19.0)	21.3 ± 1.7(19.0–23.0)	20.3(15.0–25.0)	17.9 ± 1.9(15.0–20.0)
No. of tail annuli	10.5 ± 0.7(9.0–12.0)	9.7 ± 0.5(9.0–10.0)	10.6 ± 2.0 (8–13	10.8 ± 1.3(9–13)	11.6(10.0–14.0)	12.9 ± 1.8(9–14)
Vulva to annus distance	235.4 ± 22.1(195.1–281.3)	__	__	__	__	__
Lateral field width	6.5 ± 0.2(6.1–7.0)	7.0 ± 0.6(5.9–8.1)	5.9 ± 0.5 (5.0–6.0)	6.3 ± 0.2 (6.0–7.0)	__	6.4 ± 0.6(5.5–7.5)
Lip width	7.2 ± 0.7(6.3–8.4)	6.9 ± 0.4(6.3–7.8)	6.3 ± 0.2 (6.0–6.5)	6.5 ± 0.2 (6.0–7.0)	__	6.8 ± 0.4(6.5–7.5)
Lip height	4.0 ± 0.6(3.5–5.7)	4.1 ± 0.3(3.7–4.6)	3.7 ± 0.1 (3.5–4.0)	4.2 ± 0.4 (4.0–5.0)	__	4.0 ± 0.3(3.8–4.4)

All measurements are in µm and in the form of mean ± SD (range)

n Number of specimens measured, L Body length, a Body length/greatest body width, b Body length/length from the lips to the junction of esophageal gland and intestine, b’ Body length/ length from the lips to esophageal gland end, c Body length/ tail length, c’ Tail length/tail diameter at anus, V Distance of vulva from the lips × 100/body length, DGO Distance between dorsal esophageal gland opening and stylet knobs, O DGO from stylet base × 100/Stylet length, m Conus length × 100/Stylet length

Female; Habitus spiral. (Fig. 1 A). Lip region hemispherical, with 4–5 annules (Fig. 1 B, C, D). Stylet robust, with rounded knobs that varied little in shape, 2–3 µm high and 4–6 µm wide (Fig. 1 B, C, D). Median pharyngeal bulb oval to rounded (Fig. 1 E, I). An excretory pore was immediately posterior to the hemizonid (Fig. 1 E). Two genital branches, both functional, outstretched (Fig. 1 F, G). Lateral field with four longitudinal lines (Fig. 1 H), absence of areolation in the tail region (Fig. 1 O, P). Inner lateral field incisures in tail region mostly fused distally into a Y-shaped configuration (Fig. 1 O, P). Pharyngeal glands overlapped the intestine ventrally (Fig. 1 E, I). Measurement of anal body diameter ranged from 12.7 to 14.4 µm (Fig. 1 K, L, M, N). Tail longer than the anal body diameter, with 6–13 ventral annuli, ending in a pronounced ventral projection, usually rounded terminally, without a mucro (Fig. 1 K, L, M, N). Tail tip without annulation or indistinctly annulated.

Male; Not observed.

Remarks: The specimens were identified as *H. microlobus* because of the presence of the lateral field not areolated on the tail, inner incisures of the lateral field fused distally for about two annuli in a Y-shaped pattern and tail projection not annulated. The several characteristics were consistent with the description of Siddiqi (1972) [23]. So, taking into consideration the results of our morphological and morphometric data, we consider the population as representatives of *H. microlobus* rather than *H. pseudorobustus* or another taxon. In this study, the morphological characteristics of the population of *H. microlobus* collected in this study were compared with data of Mwamula et al. (2020), Yan et al. (2017) and Subbotin et al. (2015) [18, 19, 22]. In comparison with the materials studied by Mwamula et al. (2020), the L (679.7 µm vs 732.5 µm), c (33.1 vs 37.7) and max body diameter values (25.4 µm vs 28.6 µm) for female nematodes were relatively smaller; the pharynx length (144.3 µm vs 123.6 µm) of measurements and m value (48.8 vs 43.7) were relatively larger [23]. In comparison with the North Dakota specimens studied by Yan et al. (2017), they differed in tail annules (10.0–14.0 vs 9.0–12.0) and a value (21.4–27.1 vs 25.6–28.1) [19]. Compared with the California specimens studied by Subbotin et al. (2015), the body length (679.7 µm vs 739.0 µm), anterior end to excretory pore (110.0 µm vs 128.0 µm) and stylet length (25.6 µm vs 28.9 µm) were relatively smaller; and Compared with the China specimens studied by Subbotin et al. (2015), the body length (679.7 µm vs 622.0 µm), b value (5.3 vs 4.7), Anterior to median bulb length (78.0 µm vs 58.0 µm), and tail length (20.7 µm vs 17.0 µm) were relatively larger [20].

### Molecular characterization and phylogenetic relationships of *H. microlobus*

**Fig. 2:**
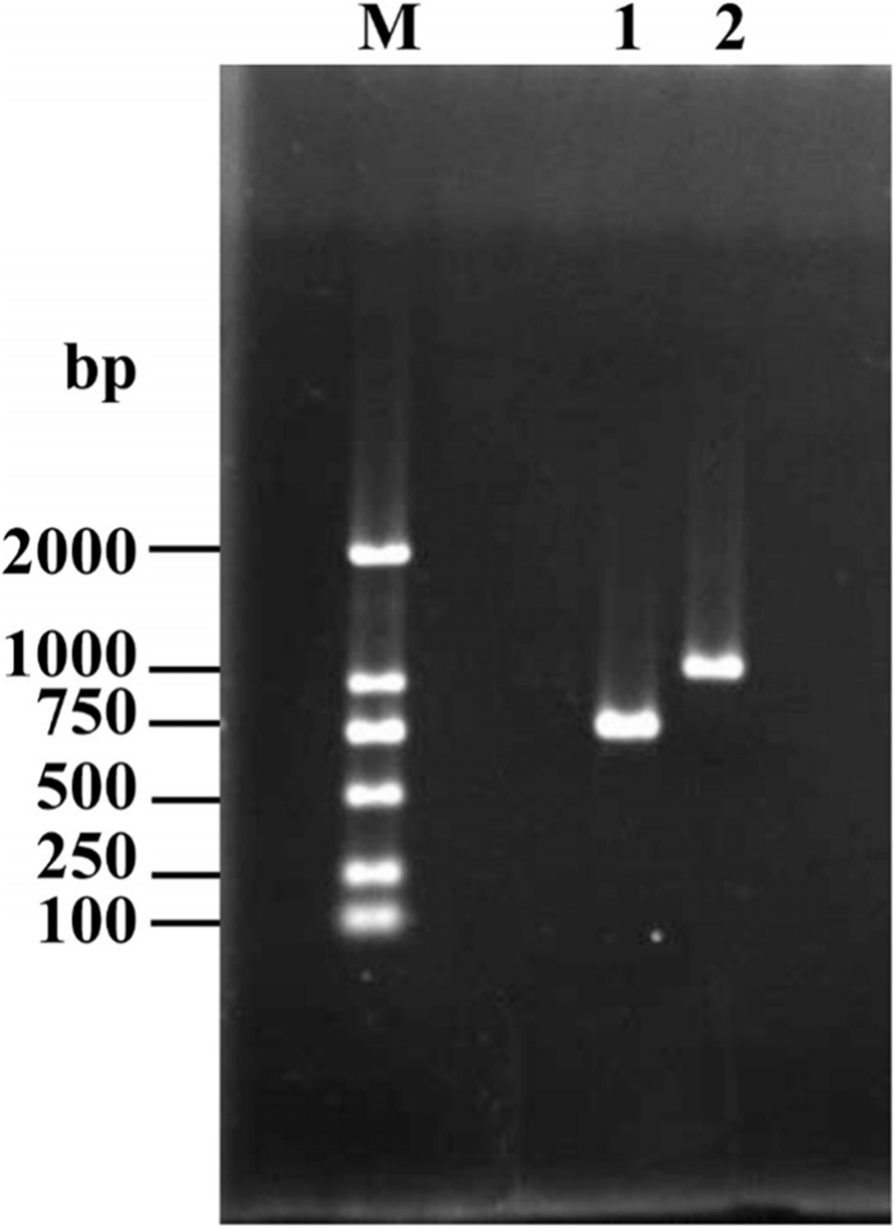
PCR amplification of the D2-D3 region of 28S rRNA and the ITS rRNA gene of *Helicotylenchus microlobus.* M: DL2000 Marker. 1: 28S. 2: ITS

The primers TW81/AB28 and D2A/D3B were used to amplify the ITS and D2-D3 regions, respectively, of the 28S rRNA gene sequences of *H. microlobus*. The amplified PCR products were 1105 bp and 787 bp in length, respectively (Fig. 2). The obtained ITS sequences and the D2-D3 region of 28S rRNA sequences in this study were submitted to GenBank database. The ITS rRNA gene sequences obtained in this study (GenBank accession No. MZ2708013) showed 99.17%-99.89% similarity with *H. microlobus* sequences from the California populations (KM506859 and KM506860) and the Korea populations (MN764342 and MN764343). The obtained D2-D3 region of the 28S rRNA gene sequences of *H. microlobus* (MZ2707554) showed 99.2%-100% similarity with *H. microlobus* sequences from the Korea populations (MN764328, MN764324, MN764323 and MN7643253) and the Island populations (MG770481).

**Fig. 3:**
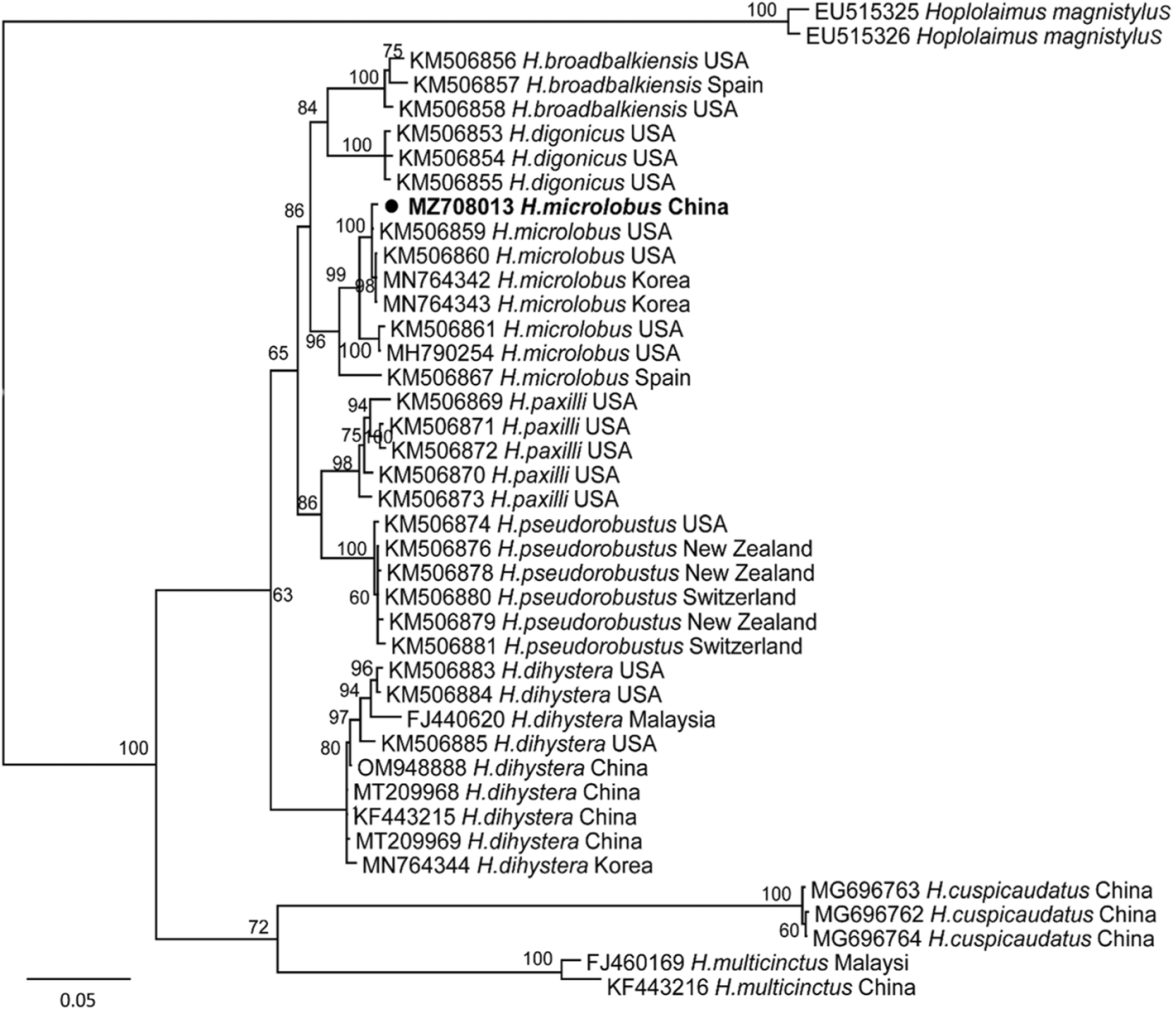
Bayesian tree of *Helicotylenchus* as inferred from ITS rRNA gene sequences under GTR + I + G model. Posterior probabilities more than 50% are given for appropriate clades. Newly obtained sequence is indicated in bold font

**Fig. 4:**
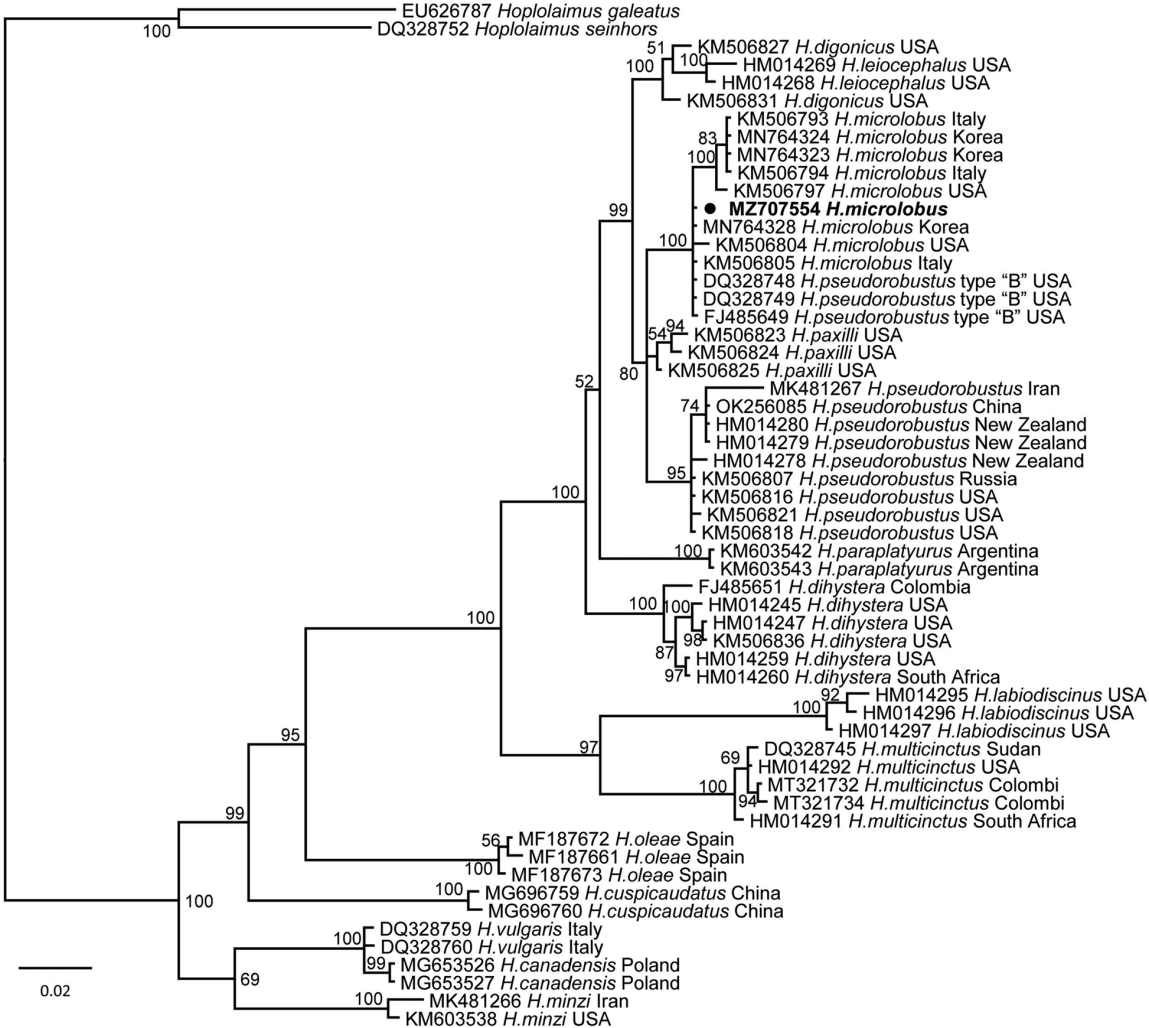
Bayesian tree of *Helicotylenchus* as inferred from the D2-D3 region of 28S rRNA gene sequences under GTR + I + G model. Posterior probabilities more than 50% are given for appropriate clades. Newly obtained sequence is indicated in bold font

Phylogenetic analysis within the genus *Helicotylenchus* based on ITS rRNA gene sequences was performed and contained 41 sequences with 954 positions in length. The 50% majority rule consensus tree inferred from the ITS data set by Bayesian analysis is shown in Fig. 3. The phylogenetic tree showed that the newly obtained sequence for *H. microlobus* (MZ2708013) formed a 100% supported clade with other *H. microlobus* population. Phylogenetic analysis within the genus *Helicotylenchus* based on the D2-D3 region of the 28S rRNA gene contained 57 sequences with 557 positions in length. The 50% majority rule consensus tree inferred from the 28S data set by Bayesian analysis is shown in Fig. 4. This phylogenetic tree indicated that the newly obtained sequence for *H. microlobus* (MZ2707554) formed a highly supported clade with *H. microlobus* and *H. pseudorobustus* type B (*pp* = 100%).

### Effects of temperature on the reproduction rate (Rr) of *H. microlobus*

**Fig. 5:**
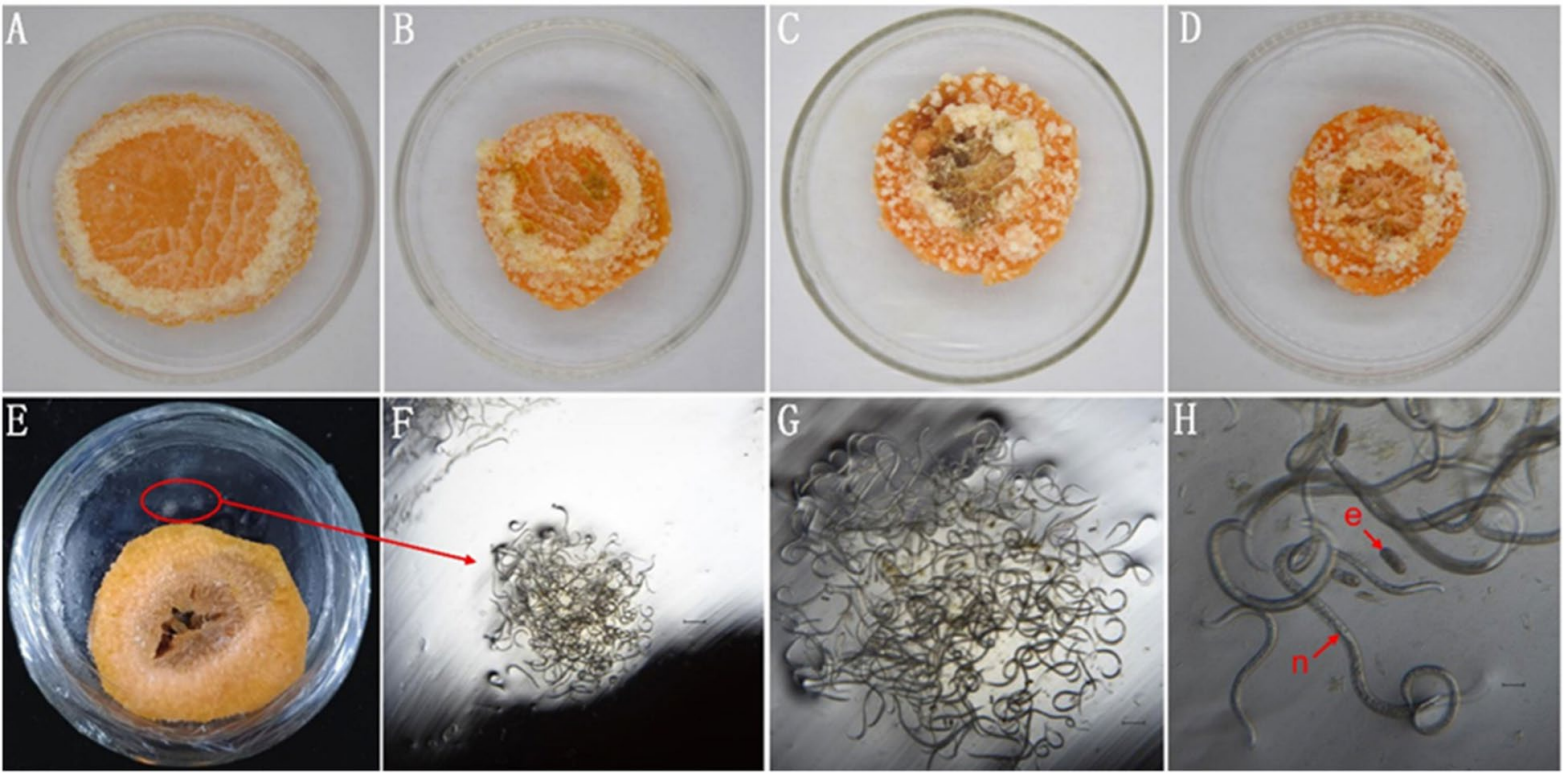
The carrot disks with disease symptoms and nematode populations at 25, 27.5 and 30 °C after 90 days. **A**: control without nematodes; **B-D**: the carrot disks with disease symptoms at 25 ℃, 27.5 ℃ and 30 ℃, respectively; **E**: nematode population; **F–H**: microscopic images of nematode populations; e = egg; n = nematode; Scale bars: F: 400 µm; G: 200 µm; H: 25 µm

At 90 days after inoculation with 30 females, reproduction occurred on each carrot disk, and the Rr reached 232, 406, and 53 at 25, 27.5 and 30 °C, respectively (Table 2). The nematode Rr at 27.5 °C was significantly higher than that at 25 and 30 °C (*P* < 0.05). In addition, when incubated at 25 and 27.5 °C for 90 days, *H. microlobus* gathered on the surface of the Petri dish (Fig. 5), and the number of nematodes reached 6947(35.9% females, 34.4% juveniles, 29.7% eggs and no males) and 12,190(34.0% females, 29.9% juveniles, 36.1% eggs and no males), respectively (Fig. 5) (Table. 2). At 90 days after inoculation with 30 females, the carrot disks presented obvious infection symptoms and turned brown or dark-brown. These results demonstrate that the carrot disk method is suitable for the culture of *H. microlobus.*

**Table 2:** Effect of temperature (Tm) on the reproduction of *Helicotylenchus microlobus* on carrot callus 90 days after inoculation with 30 females ^x^

Tm (℃)	Females	Juveniles	Eggs	*Pf *^*y*^	*Rr* = *Pf/Pi *^*z*^
25	2496 ± 283.4 b	2390 ± 376.1 b	2061 ± 400.8 b	6947 ± 718.4 b	232 ± 23.9 b
27.5	4141 ± 459 a	3646 ± 410 a	4403 ± 510.1 a	12,190 ± 881.9 a	406 ± 29.4 a
30	514 ± 95.6 c	499 ± 65.2 c	590 ± 113.8 c	1603 ± 236.9 c	53 ± 7.9 c

^x^Different letters (a, b, c) in columns indicate significant differences (*P* < 0.05) according to the Duncan’s multiple range test. Each number is the mean of five replicates. Values represent the mean ± standard error

^*y*^*Pf* = final number of nematodes, including eggs and vermiform stages

^z^Reproduction rates of nematodes (Rr) = *Pf*/initial number of nematodes (*Pi*)

## Discussion

Plant-parasitic nematodes are some of the most important pathogens of tomato. *Meloidogyne*, *Helicotylenchus, Pratylenchus*, *Tylenchorhynchus* and *Aphelenchoides* can infect tomato roots and cause severe economic losses [24]. Different *Helicotylenchus* have been reported from the rhizosphere soil of tomato in many countries. For example, in Turkey, *H. digonicus*, *H. tunisiensis* and *H. varicaudatus* have been reported from tomato rhizosphere soils [25]. Kim et al. (2014) reported that *H. dihystera* can suppress the growth of tomato and this species was also reported from tomato in China [26]. *H. thornei* Roman, 1965 is also described from soil around the roots of tomato in Ludhiana, India [27].

The genus *Helicotylenchus* known to contain various species complexes that normally exhibit similar diagnostic characteristics [18]. As intraspecific variability in characters may be influenced by environmental conditions, species delineation within the genus is not always easy [28]. This adds to identification problems and can leads to some misidentifications within the genus, due to lack of consensus among different taxonomists on the validity of some species [18, 29]. Therefore, biochemical and molecular information are more and more important in nematode taxonomy, however, these approaches should still be integrated with morphological characteristics [30].

*H. microlobus* was considered as a junior synonym of *H. pseudorobustus* by Sher (1966) because of their indistinguishable morphological characteristics, and this propose was accepted by Sauer and Winoto (1975), Fortuner (2018), and Divsalar et al. (2020) but was not agreed with by Siddiqi (1972, 2000), Subbotin et al. (2015) and Mwamula et al. (2020) [6, 18, 22, 23, 29, 31, 32]. Siddiqi (1972) proposed that *H. microlobus* differed from *H. pseudorobustus* by the non areolated lateral field in tail region (areolated in *H. pseudorobustus)*, and inner lateral lines fusion as Y-shaped in the tail region (M- or U-shaped in *H. pseudorobustus*) [18, 23]. In this study, the morphological characteristics of this population were consistent with the characteristics of *H. microlobus* described by Siddiqi (1972), Subbotin et al. (2015) and Mwamula et al. (2020) [18, 22, 23]. When compared with *H. microlobus* species, the obtained ITS rRNA sequence showed 99.17%-99.89% homology with both *H. microlobus* sequences from California (KM506859 and KM506860), Korea (MN764342 and MN764343) and Spain (KM506862). In contrast, it had only 93% sequence identity with isolates of *H. pseudorobustus* (KM506835) from California. And, the obtained 28S rRNA sequence showed 99.2%-100% homology with both *H. microlobus* sequences from Korea (MN764328, MN764324, MN764323 and MN7643253) and Island (MG770481). In contrast, it had only 91% sequence identity with isolates of *H. pseudorobustus* (KU722387) from Iran. In addition, the results of the phylogenetic analysis of the ITS rRNA gene region showed that *H. microlobus* and *H. pseudorobustus* were clearly divided into two different clades; the results of the phylogenetic analysis of the 28S rRNA gene D2-D3 gene region showed that *H. pseudorobustus* type B (DQ328748; DQ328749; FJ485649) were clustered together with *H. microlobus*. Subbotin et al. (2011, 2015) proposed that a species group complex for *H. pseudorobustus* includes at least *H. pseudorobustus* type A and *H. pseudorobustus* type B, according to morphological and molecular analyses. According to the results of Subbotin et al. (2015), *H. pseudorobustus* type B should be *H. microlobus* and phylogenetic relationships of *H. pseudorobustus* type B (DQ328748; DQ328749; FJ485649) were clustered together with *H. microlobus*. Therefore, our results were consistent with the results of analysis of *Helicotylenchus* species by Subbotin et al. (2011, 2015). Taking into consideration the results of our integrative morphological and molecular analyses of the *H. microlobus* population, we considered the populations as representatives of *H. microlobus* rather than *H. pseudorobustus*. This is the first report of *H. microlobus* from tomato in China using morphological and molecular characterization.

Obtaining a large number of plant-parasitic nematodes is very important because many types of studies can be performed with these nematodes, such as pathogenicity tests and biological and genetic studies [21]. For a long time, researchers have been looking for methods to culture plant-parasitic nematodes using plant tissues [24, 25]. Some culture methods have been successfully employed, and there are great differences in these methods. For example, *Aphelenchoides besseyi*, *Ditylenchus destructor* and *Bursaphelenchus xylophilus* can be cultured on certain fungi [33–35]. *A. ritzemabosi* and *D. dipsaci* can reproduce rapidly on alfalfa tissue [36]. The carrot callus method is suitable for culturing *A. ritzemabosi, A. besseyi*, *R. similis*, and most species of *Pratylenchus* [37, 38]. Over the years, some researchers tried to culture certain *Helicotylenchus* species in vitro, but failed. For example, Brown and Vessey demonstrated that *H. multicinctus* failed to survive when cultured on banana fruit callus [39]; Khera and Zuckerman cultured *H. erythrinae* on tissues of carrot, sweet potato, tobacco and tomato, but nematodes all failed to reproduction on these tissues [40]; Kagoda et al. reported that *H. multicinctus* failed to rear on carrot discs [41]. One *Helicotylenchus* species and *H. dihystera* have been reported to successfully produce progenies after inoculating single female in host-plants pots [42, 43]. However, the method culturing the *Helicotylenchus* species in host-plants pots was susceptible to contamination, time-consuming and difficult to separate the progeny from the pots. Sterile carrot disks are usually regarded as a relatively low-cost, straightforward and efficient method for culturing some nematodes that results in greater nematode multiplication compared with other methods [21]. Our study is the first to successfully establish an artificial culture method for *H. microlobus* on carrot disks. The succcesful rearing of *H. microlobus* make other related biological studies of *H. microlobus* possible.

Plant growth inhibition has been reported associated with several *Helicotylenchus* species that have also increased secondary infections of fungal pathogens [44]. Studies have shown that when *H. dihystera* and *Pseudomonas solanacearum* present together, tomato wilting was much more serious than when present alone, and when *H. dihystera* associated with *P. caryophylli*, carnation wilting was also significantly increased [12, 45]. Therefore, whether there is such a synergistic relationship between *H. microlobus* and certain bacteria that may led to more serious disease on tomato should be further investigated.

## Conclusions

In our study, both morphological and molecular analyses showed that the species of the *Helicotylenchus* population was *H. microlobus*. This is the first report of *H. microlobus* from tomato in China. We established that the carrot disk method is suitable for rearing of *H. microlobus* on carrot disks, and the optimum temperature for *H. microlobus* culture on carrot disks was 27.5 °C, and the Rr reached 406 after 90 days of inoculation with 30 females.

## Methods

### Nematode collection

In August 2019, five samples of roots and corresponding rhizosphere soils were collected from a tomato (*cv.* Maohong 801) field near Zhuma village in Tongxu County of Kaifeng city, Henan Province, China. Each sample consisted of at least five sub samples collected from patches of poor growth. Samples were placed in plastic bags, sealed transported to the laboratory in refrigerated counter and stored at 16 to 18 °C [46]. Nematodes were extracted from tomato soil and macerated root samples using the modified Baermann funnel method [47].

### In vitro rearing of nematodes

Carrot disks were prepared in the following way. The surface of a carrot was sterilized with 95% ethanol. The carrot was peeled with a sterile knife and cut into 6 mm thick disks. Each carrot tissue was placed in a 6-cm-diameter petri dish and maintained at 25 °C for 15 days for later use. One female from the collected specimens was selected and surface sterilized for 6 h with 0.3% streptomycin sulfate, and then transferred to carrot disks. and the petri dishes were sealed with parafilm and then kept in a darkened incubator at 25 ℃for 15 weeks. After that, the single female *Helicotyenchus* population cultured on carrot disks were used for morphological and molecular analysis.

### Morphological identification

Nematodes were heat-killed in water, fixed in FG (formalin: glycerin: water = 10:1:89), and processed to glycerin by the formalin glycerin method [47]. Photomicrographs and morphometric data of the *Helicotylenchus* specimens were obtained using a Nikon Eclipse Ti-S microscope (Japan). Images of key morphological features were processed using Photoshop CS5. The de Man formula was used for measurements [30]. All measurements are expressed in micrometers (μm) [48].

### Molecular characterization and phylogenetic relationships

DNA from one *Helicotylenchus* specimen was extracted using proteinase K-based lysis [49]. The rRNA-internal transcribed spacer (ITS) region and the D2-D3 region of the 28S rRNA gene were amplified with primers TW81/AB28 (5´-GTTTCCGTAGGTGAACCTGC-3´/5´-ATATGCTTAAGTTCAGCGGGT-3´) [50] and primers D2A-D3B (5´-ACAAGTACCGTGAGGGAAAGTTG-3´/5´TCGGAAGGAACCAGCTACTA-3´) [51], respectively. The processes of PCR amplification and cloning were carried out according to the method of Wang et al. [52]. Sequencing was performed by Sangon Biotech Co. Ltd. (Shanghai, PR China). The newly obtained DNA sequences were submitted to the NCBI GenBank (https://submit.ncbi.nlm.nih.gov/subs/genbank/) database.

The obtained ITS region and D2-D3 region of the 28S sequences were subjected to multiple alignment using the MAFFT Q-INS-i algorithm [53] with other *Helicotylenchus* species sequences published in the NCBI GenBank database. Outgroup taxa were selected based on a previous study [54]. Sequence datasets were analysed with Bayesian inference (BI) using MrBayes 3.2.6 [55] under the best-fit model of GTR + G + I, according to Akaike Information Criteria [56]. BI analysis for each gene was run with a random starting tree and four Markov chains for 1 × 10^6^ generations. The Markov chains were sampled at intervals of 100 generations. After discarding burns in samples, the remaining samples were used to generate a 50% majority rule consensus tree. Posterior probabilities (pp) are given on appropriate clades.

### Reproduction tests

Using the carrot disk method, experiments were undertaken to determine the reproductive potential of *H. microlobus* and also to determine the optimum temperature for rearing *H. microlobus* on carrot disks. In the experiment, 30 females were surface sterilized for 6 h with 0.3% streptomycin sulfate and then transferred to a carrot disk in a petri dish. The petri dishes were sealed with parafilm and incubated in a darkened incubator at 25, 27.5, and 30 °C, respectively. The number of nematodes and reproduction rate (Rr = final number of nematodes/initial number of nematodes) on each carrot disk were determined at 90 days after inoculation.

A maceration method was used to collect the nematodes [46]: the carrot disks were placed in sterile water and macerated in a blender. The suspension was poured through 0.250-mm and 0.150-mm-pore sieves. The nematode suspension was collected in a beaker and the carrot tissues discarded. The nematode suspension was left to settle for at least 4 h and the supernatant was removed by pipettor. The nematodes were enumerated to determine whether *H. microlobus* reproduction had occurred and the Rr (final number of nematodes/initial number of nematodes) determined. There were five replicates for each experiment, and each experiment was conducted twice. Data on *H. microlobus* cultured on carrot disks under different temperatures were subjected to a one-way analysis of variance (ANOVA), and differences were tested using Duncan’s multiple range test (DMRT) at the 5% significance level using SPSS software (ver. 13.0; SPSS Inc., Chicago). The result of this experiments are given in Table.

## Data Availability

The datasets supporting the conclusions of this article are available in the GenBank repository (https://www.ncbi.nlm.nih.gov/). The newly obtained rRNA sequences in this study were deposited in the NCBI GenBank (https://submit.ncbi.nlm.nih.gov/subs/genbank/) database for a BLAST search (accession no. MZ2708013 and MZ2707554).
